# Supplementary material for: Unraveling the Roles of miR-204-5p and HMGA2 in Papillary Thyroid Cancer Tumorigenesis
Source: Int J Mol Sci. 2023 Jun 28;24(13):10764. doi: 10.3390/ijms241310764 (PMC10341554; doi:10.3390/ijms241310764)
Supplement: Supplementary file 1 [file ijms-24-10764-s001.zip › Supplementary material final.pptx]

## Slide 1
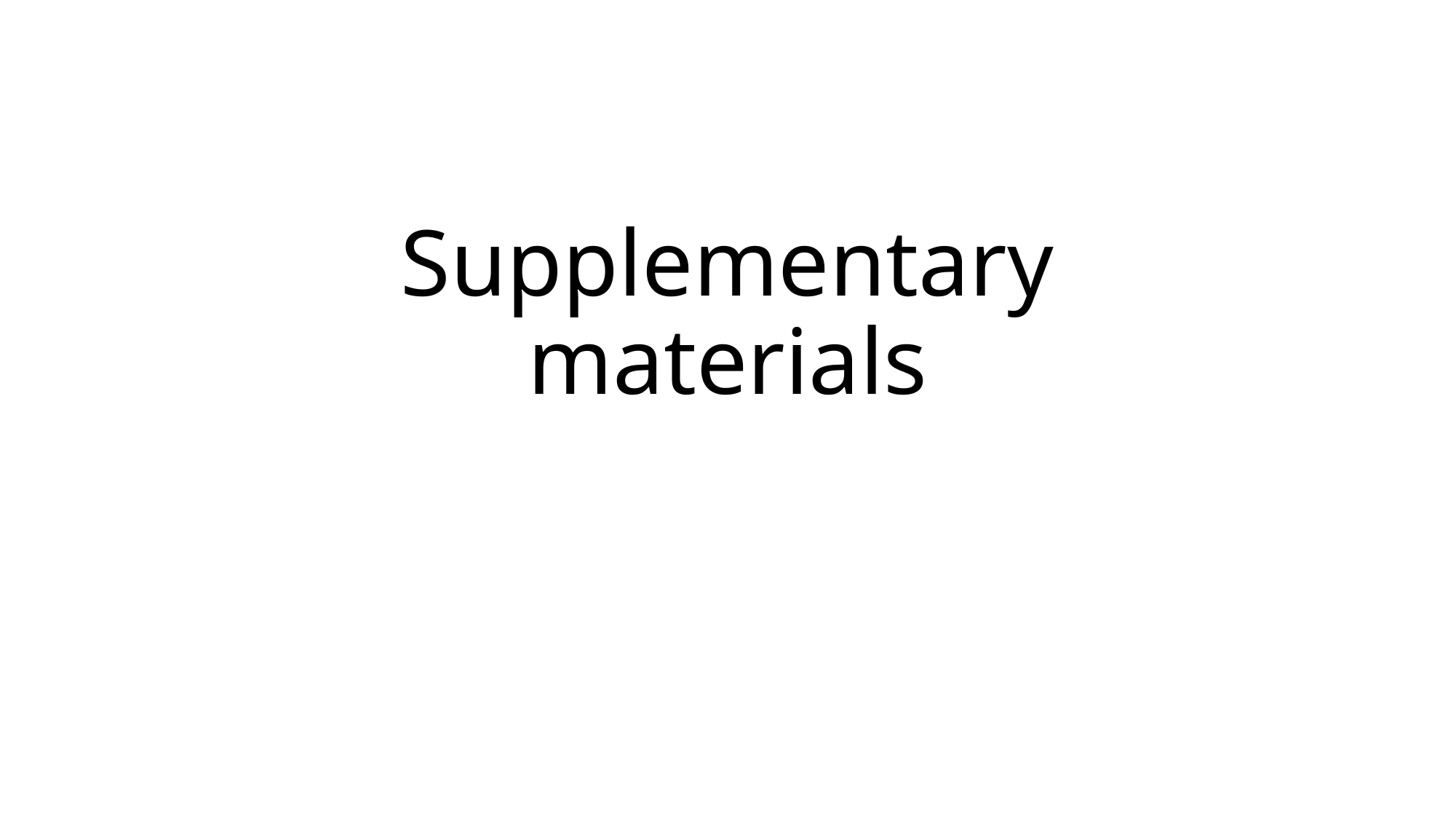

# Supplementary materials

## Slide 2
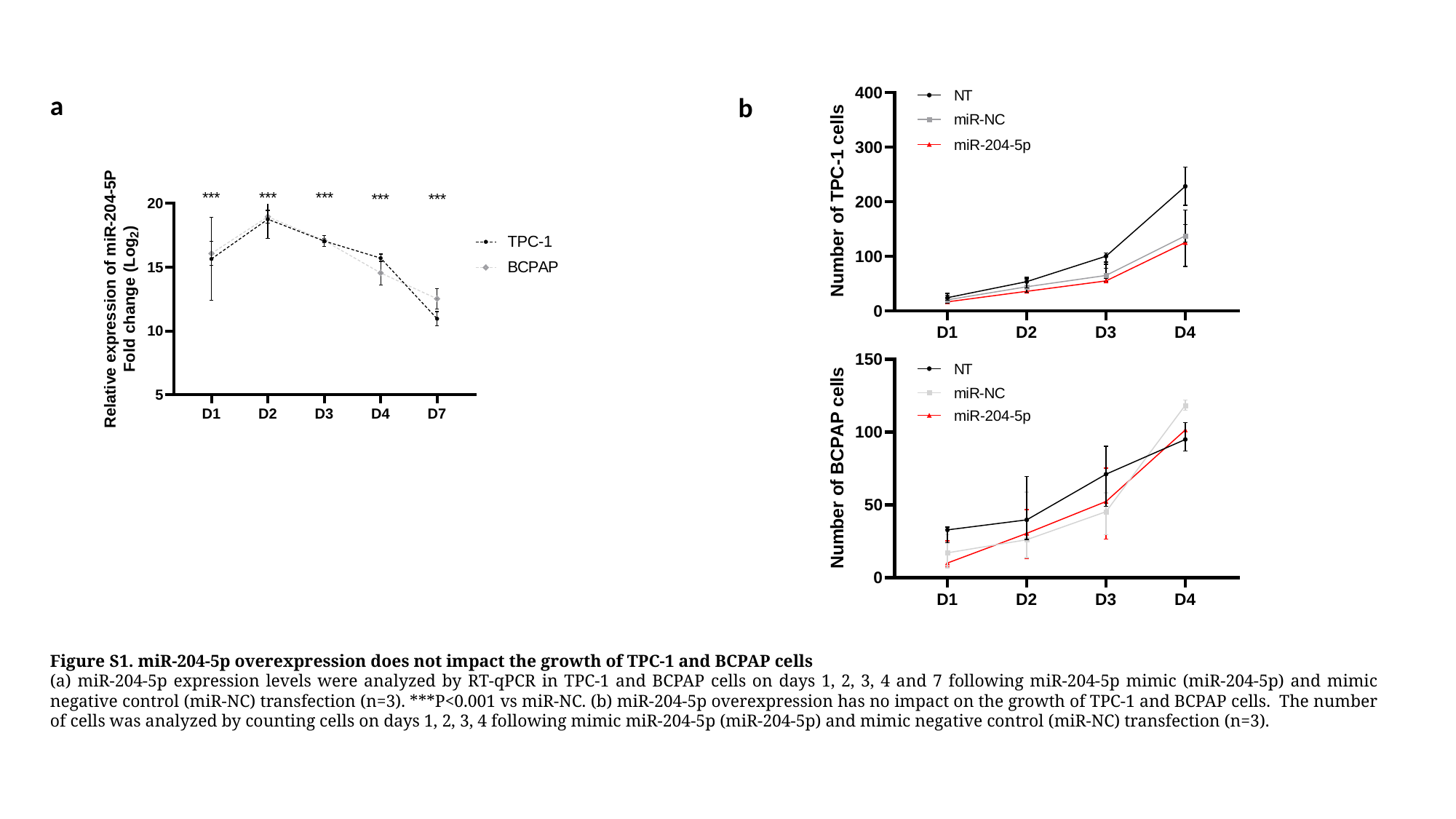

a
b
Figure S1. miR-204-5p overexpression does not impact the growth of TPC-1 and BCPAP cells
(a) miR-204-5p expression levels were analyzed by RT-qPCR in TPC-1 and BCPAP cells on days 1, 2, 3, 4 and 7 following miR-204-5p mimic (miR-204-5p) and mimic negative control (miR-NC) transfection (n=3). ***P<0.001 vs miR-NC. (b) miR-204-5p overexpression has no impact on the growth of TPC-1 and BCPAP cells. The number of cells was analyzed by counting cells on days 1, 2, 3, 4 following mimic miR-204-5p (miR-204-5p) and mimic negative control (miR-NC) transfection (n=3).

## Slide 3
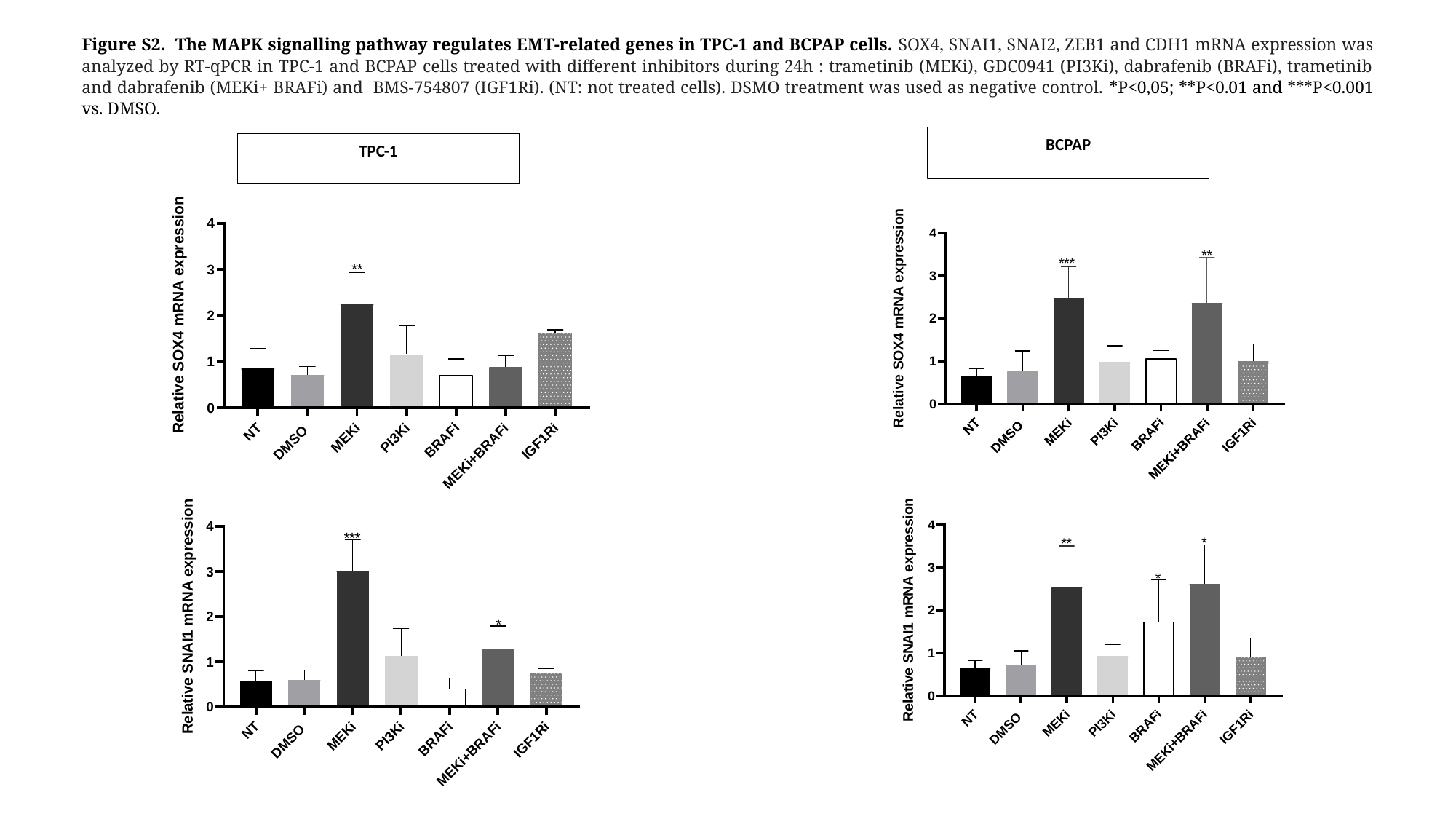

Figure S2. The MAPK signalling pathway regulates EMT-related genes in TPC-1 and BCPAP cells. SOX4, SNAI1, SNAI2, ZEB1 and CDH1 mRNA expression was analyzed by RT-qPCR in TPC-1 and BCPAP cells treated with different inhibitors during 24h : trametinib (MEKi), GDC0941 (PI3Ki), dabrafenib (BRAFi), trametinib and dabrafenib (MEKi+ BRAFi) and BMS-754807 (IGF1Ri). (NT: not treated cells). DSMO treatment was used as negative control. *P<0,05; **P<0.01 and ***P<0.001 vs. DMSO.
BCPAP
TPC-1

## Slide 4
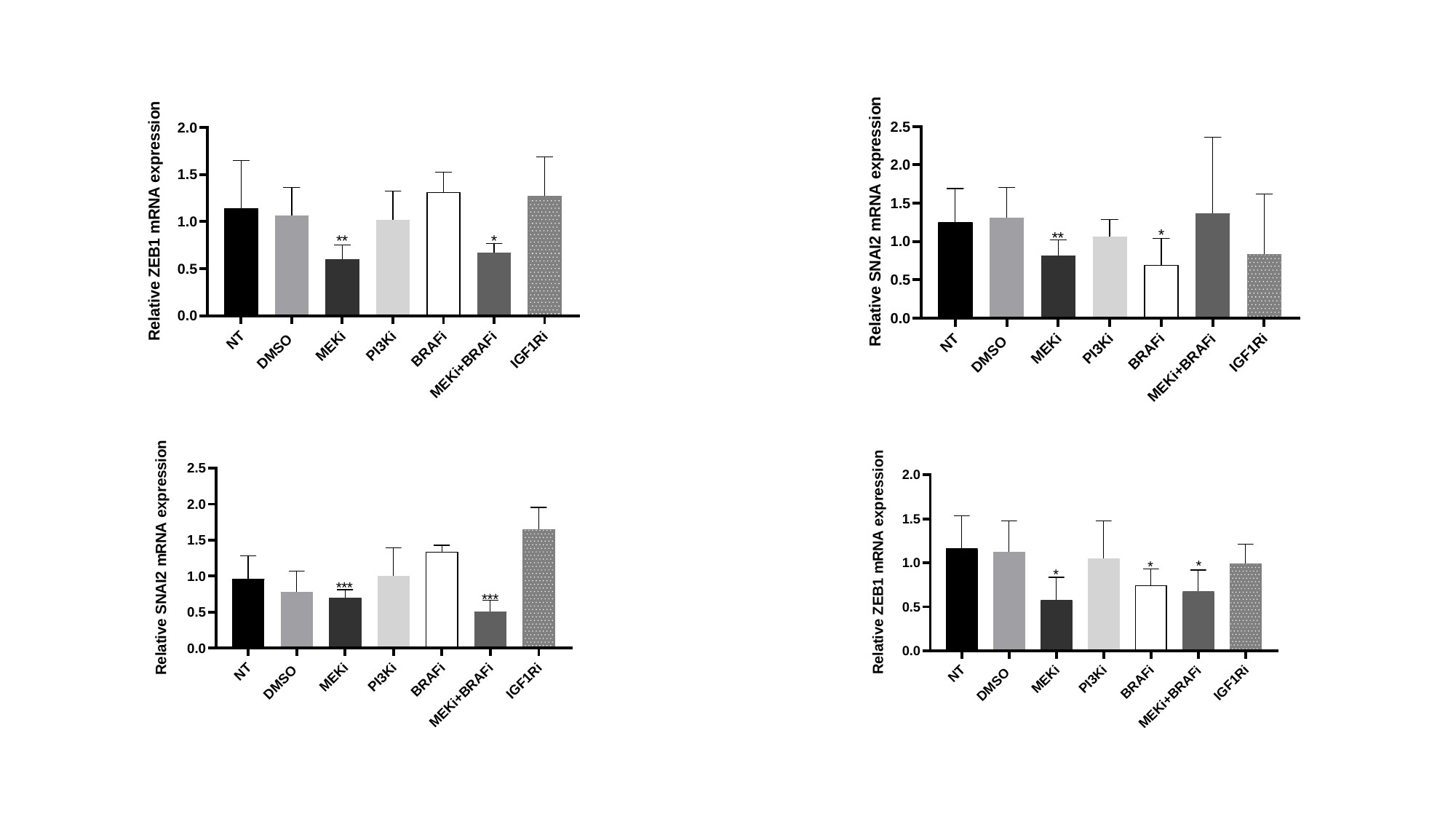

## Slide 5
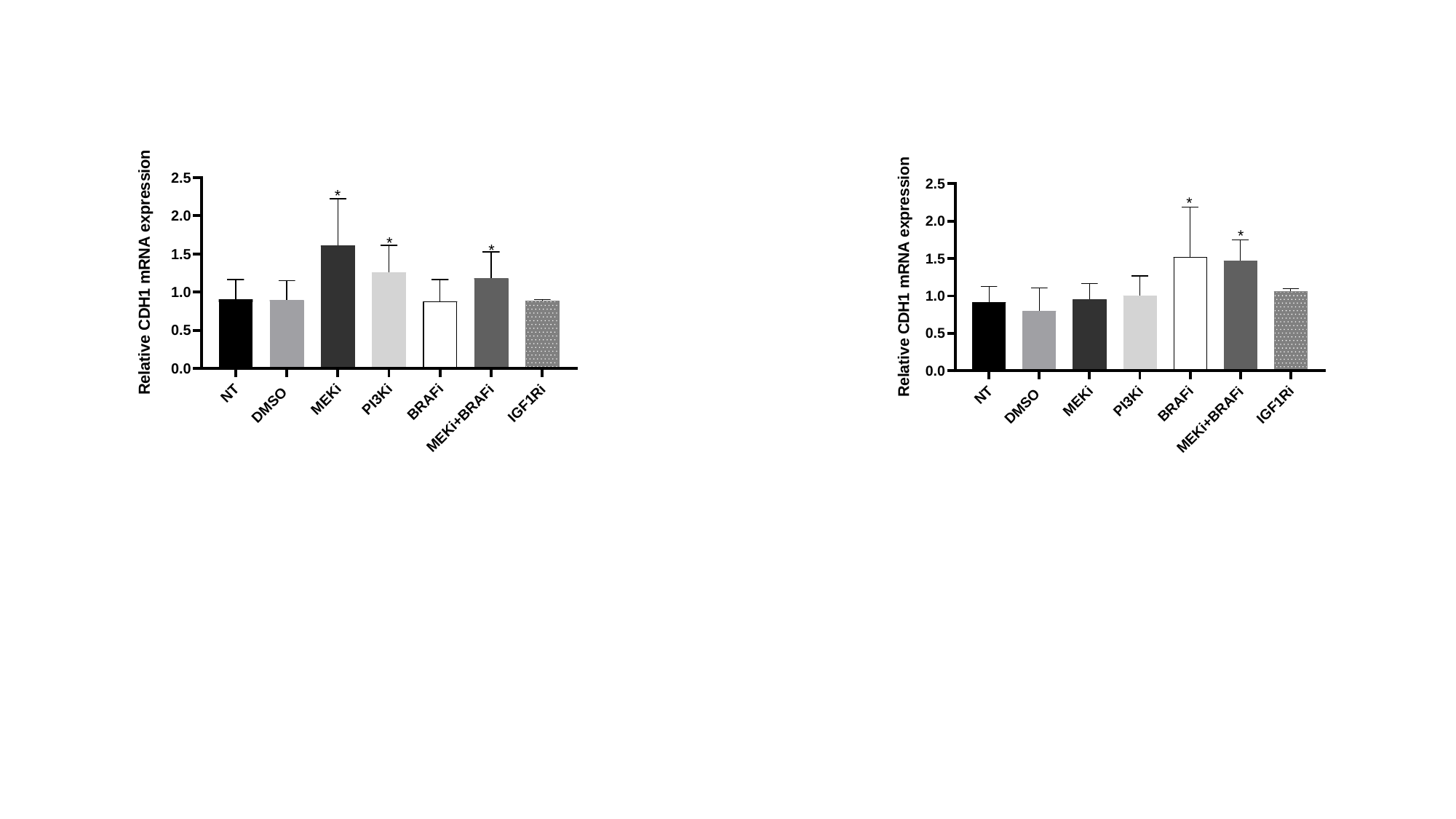

## Slide 6
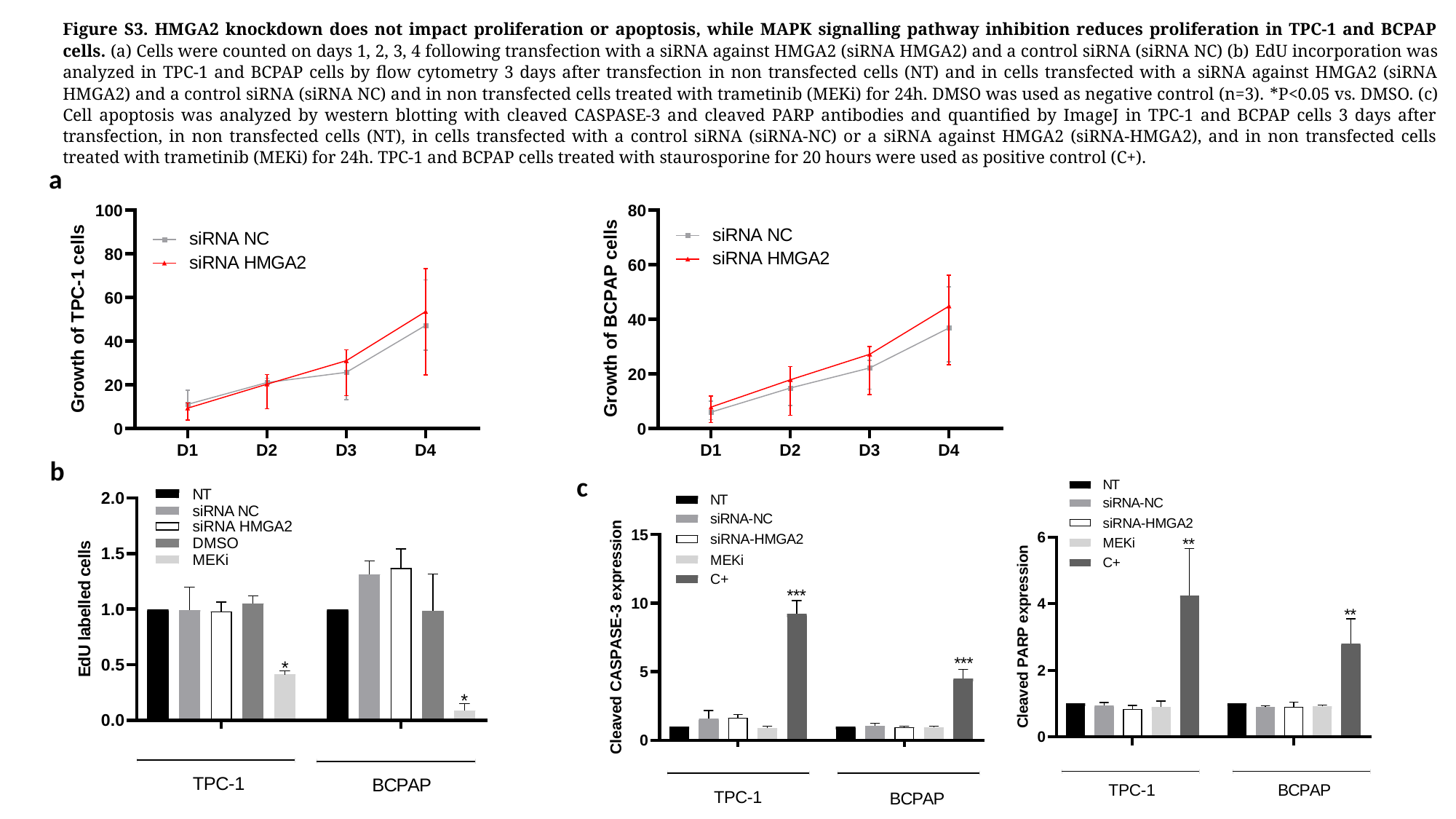

Figure S3. HMGA2 knockdown does not impact proliferation or apoptosis, while MAPK signalling pathway inhibition reduces proliferation in TPC-1 and BCPAP cells. (a) Cells were counted on days 1, 2, 3, 4 following transfection with a siRNA against HMGA2 (siRNA HMGA2) and a control siRNA (siRNA NC) (b) EdU incorporation was analyzed in TPC-1 and BCPAP cells by flow cytometry 3 days after transfection in non transfected cells (NT) and in cells transfected with a siRNA against HMGA2 (siRNA HMGA2) and a control siRNA (siRNA NC) and in non transfected cells treated with trametinib (MEKi) for 24h. DMSO was used as negative control (n=3). *P<0.05 vs. DMSO. (c) Cell apoptosis was analyzed by western blotting with cleaved CASPASE-3 and cleaved PARP antibodies and quantified by ImageJ in TPC-1 and BCPAP cells 3 days after transfection, in non transfected cells (NT), in cells transfected with a control siRNA (siRNA-NC) or a siRNA against HMGA2 (siRNA-HMGA2), and in non transfected cells treated with trametinib (MEKi) for 24h. TPC-1 and BCPAP cells treated with staurosporine for 20 hours were used as positive control (C+).
a
b
c

## Slide 7
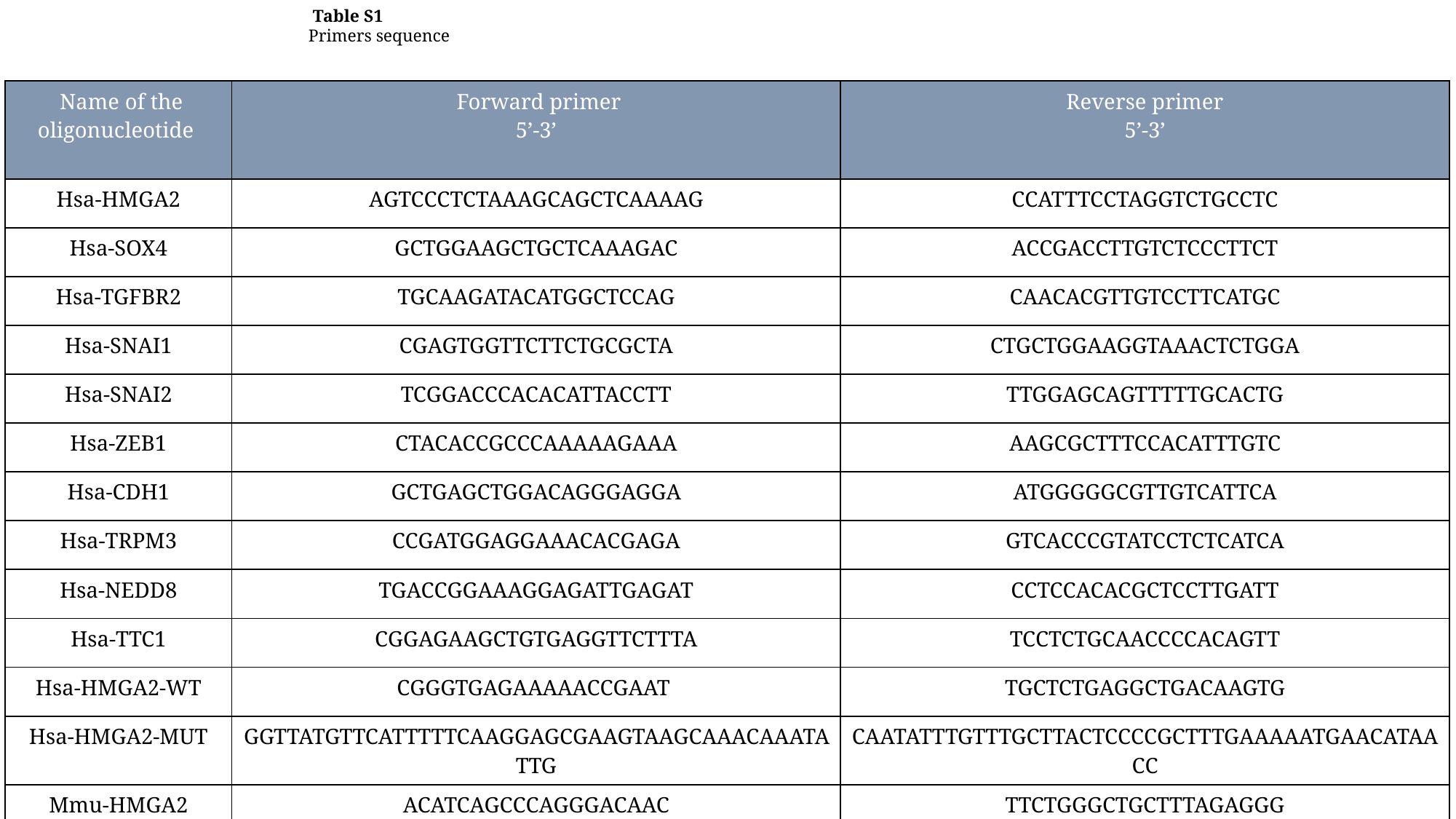

Table S1
Primers sequence
| Name of the oligonucleotide | Forward primer 5’-3’ | Reverse primer 5’-3’ |
| --- | --- | --- |
| Hsa-HMGA2 | AGTCCCTCTAAAGCAGCTCAAAAG | CCATTTCCTAGGTCTGCCTC |
| Hsa-SOX4 | GCTGGAAGCTGCTCAAAGAC | ACCGACCTTGTCTCCCTTCT |
| Hsa-TGFBR2 | TGCAAGATACATGGCTCCAG | CAACACGTTGTCCTTCATGC |
| Hsa-SNAI1 | CGAGTGGTTCTTCTGCGCTA | CTGCTGGAAGGTAAACTCTGGA |
| Hsa-SNAI2 | TCGGACCCACACATTACCTT | TTGGAGCAGTTTTTGCACTG |
| Hsa-ZEB1 | CTACACCGCCCAAAAAGAAA | AAGCGCTTTCCACATTTGTC |
| Hsa-CDH1 | GCTGAGCTGGACAGGGAGGA | ATGGGGGCGTTGTCATTCA |
| Hsa-TRPM3 | CCGATGGAGGAAACACGAGA | GTCACCCGTATCCTCTCATCA |
| Hsa-NEDD8 | TGACCGGAAAGGAGATTGAGAT | CCTCCACACGCTCCTTGATT |
| Hsa-TTC1 | CGGAGAAGCTGTGAGGTTCTTTA | TCCTCTGCAACCCCACAGTT |
| Hsa-HMGA2-WT | CGGGTGAGAAAAACCGAAT | TGCTCTGAGGCTGACAAGTG |
| Hsa-HMGA2-MUT | GGTTATGTTCATTTTTCAAGGAGCGAAGTAAGCAAACAAATATTG | CAATATTTGTTTGCTTACTCCCCGCTTTGAAAAATGAACATAACC |
| Mmu-HMGA2 | ACATCAGCCCAGGGACAAC | TTCTGGGCTGCTTTAGAGGG |
